# Renoprotective effects of dexmedetomidine against ischemia-reperfusion injury in streptozotocin-induced diabetic rats

**DOI:** 10.1371/journal.pone.0198307

**Published:** 2018-08-16

**Authors:** Seung Hyun Kim, Ji Hae Jun, Ju Eun Oh, Eun-Jung Shin, Young Jun Oh, Yong Seon Choi

**Affiliations:** 1 Department of Anesthesiology and Pain Medicine, Severance Hospital, Yonsei University College of Medicine, Seoul, Korea; 2 Anesthesia and Pain Research Institute, Severance Hospital, Yonsei University College of Medicine, Seoul, Korea; University of PECS Medical School, HUNGARY

## Abstract

**Background:**

Diabetic patients are susceptible to renal ischemia-reperfusion injury, which leads to perioperative complications. Activation of NOD-like receptor protein 3 (NLRP3) inflammasome participates in the development of diabetes, and contributes to renal ischemia-reperfusion injury. Dexmedetomidine (DEX), a highly selective α2-adrenoreceptor agonist, shows renoprotective effects against ischemia-reperfusion injury. We aimed to elucidate the effects, underlying mechanisms, and optimal timing of DEX treatment in diabetic rats.

**Methods:**

Male Sprague-Dawley rats (n = 12 per group) were randomly divided into normal-sham, diabetes-sham, diabetes-ischemia-reperfusion-control, diabetes-ischemia-reperfusion-DEX-pre-treatment, and diabetes-ischemia-reperfusion-DEX-post-treatment groups. Renal ischemia-reperfusion injury was induced in diabetic rats by occlusion of both renal arteries for 45 min, followed by reperfusion for 24 h. DEX (10 μg/kg) was administered intraperitoneally 1 h before ischemia (pre-treatment) or upon reperfusion (post-treatment). After reperfusion, renal tissue was biochemically and histopathologically evaluated.

**Results:**

DEX treatment attenuated ischemia reperfusion-induced increase in NLRP3, caspase-1, IL-1β, phospho-AKT, and phospho-ERK signaling. Moreover, oxidative stress injury, inflammatory reactions, apoptosis, and renal tubular damage were favorably modulated by DEX treatment. Furthermore, post-reperfusion treatment with DEX was significantly more effective than pre-treatment in modulating NLRP3 inflammasome, AKT and ERK signaling, and oxidative stress.

**Conclusions:**

This study shows that the protective effects of DEX in renal ischemia-reperfusion injury are preserved in diabetic conditions and may potentially provide a basis for the use of DEX in clinical treatment of renal ischemia-reperfusion injury.

## Introduction

Acute kidney injury (AKI) is a common complication during major surgeries and is associated with increased risk of chronic kidney disease and mortality [1]. Diabetes mellitus is a metabolic disorder with major complications, including microvascular and macrovascular diseases, and an important risk factor for AKI resulting in renal damage and dysfunction, as it increases susceptibility to ischemia-reperfusion (IR) injury [2].

Inflammasomes are components of immune inflammatory reactions that respond to danger signals and trigger inflammatory cascades. NOD-like receptor protein 3 (NLRP3) inflammasome has been implicated in the pathogenesis of numerous renal conditions, including AKI, diabetic nephropathy, and chronic kidney disease [3]. Activation of NLRP3 inflammasome leads to maturation and secretion of the proinflammatory cytokines interleukin (IL)-1β and IL-18, resulting in renal inflammation and fibrosis [4]. Thus, NLRP3 inflammasome may be a feasible therapeutic target for renal IR injury in diabetic patients.

Dexmedetomidine (DEX) is a highly selective and potent α2 adrenergic agonist possessing sedative, analgesic, and sympatholytic properties. In the kidney, α2 adrenoceptors are distributed widely in the proximal and distal tubules, and in peritubular vasculature. In recent animal studies, DEX has been reported to reduce the number of apoptotic tubular epithelial cells and improve tubular architecture and function following renal IR injury [5–7]. Moreover, studies in animal model of diabetes showed that DEX protected the kidney, heart, and brain against IR injury through its anti-apoptotic, anti-inflammatory, and antioxidant effects [8–10]. Despite its clinical importance in mitigating renal IR injury in surgical patients, there is lack of evidence on the renoprotective effects and related mechanisms of DEX in diabetic conditions. Therefore, in the present study, we aimed to investigate the renoprotective effects of DEX against renal IR injury in diabetic rats and to elucidate the relation between DEX and NLRP3 inflammasome. Additionally, this study attempted to identify DEX treatment regimens achieving optimal renoprotective effects in a diabetic rat renal IR injury model.

## Materials and methods

### Animal preparation

All animal experiments were approved by the Committee for the Care and Use of Laboratory Animals, Yonsei University College of Medicine (No. 2015–0129, approval date 16.06.2015), and were performed in accordance with the *Guide for the Care and Use of Laboratory Animals* published by the US National Institutes of Health [11].

Male Sprague-Dawley rats (10–12 weeks old, 250–300 g) purchased from Orient Bio Inc. (Korea) were used. Diabetes was induced by a single intraperitoneal injection of freshly prepared streptozotocin (STZ) (85 mg/kg) dissolved in 100 mM sodium citrate buffer (pH 4.5), and operations were performed 2 weeks after STZ administration. Blood sugar concentration was determined using tail blood samples. The rats were housed in a temperature and light-controlled animal facility with alternating 12-hour light/dark cycles and *ad libitum* access to standard laboratory chow and water.

General anesthesia was induced using xylazine (Rompun, 10 mg/kg) and tiletamine + zolazepam (Zoletil 50, Virbac Korea, 30 mg/kg). The rats were intubated with a 16-gauge (G) catheter and artificially ventilated (Harvard Apparatus 683, Holliston, MA, USA) at 30–35 cycles/min. The right femoral artery was cannulated to monitor mean arterial pressure (MAP) and collect blood. Heart rate (HR) was monitored by subcutaneous stainless-steel electrodes connected to the power lab system (ML845 PowerLab with ML132; AD Instruments, Colorado Springs, CO, USA). Body temperature was maintained at approximately 37°C using a heating pad and continuously monitored throughout the experiment.

### Experimental models and study groups

The rats were randomly divided (12 rats per group) into normal-sham (N-sham), diabetic-sham (D-sham), diabetic IR-control (D-IRC) receiving normal saline, diabetic IR-DEX pre-treatment (D-DEXpre), and diabetic IR-DEX post-treatment (D-DEXpost) groups. We used a previously described rodent model of renal IR injury [12]. In brief, both rat renal arteries were clamped for 45 min with non-traumatic microvascular clamps followed by 24 h of reperfusion. Ischemia and reperfusion were confirmed by visually inspecting the kidneys. MAP and HR were continuously monitored and recorded during the procedures (baseline, during ischemia, and after reperfusion). All diabetic rats underwent midline incision to expose both kidneys. To examine DEX renoprotective effects, DEX (Precedex, Pfizer, 10 μg/kg) was administered 1 h before ischemia or upon reperfusion, whereas the control group received an equivalent amount of normal saline. Initially, we tested the efficacy of various DEX doses (10, 20, 50, and 100 μg/kg) 1 h before ischemia and found that 10 μg/kg yielded optimal renoprotective effects against renal tubular damage. The IR-control groups received equivalent amounts of normal saline via tail vein. Blood glucose concentration >200 mg/dl was considered high glucose level [13]. Blood glucose concentrations were determined at baseline, before ischemia, upon reperfusion, and 24 h later. The kidneys were collected after 24-h reperfusion.

### Periodic acid-Schiff staining

Tissue samples for histopathological examination were taken from the left kidney (including the ischemic zone) after 24-h reperfusion, fixed in 10% buffered formalin, and embedded in paraffin. Paraffin-embedded kidney tissues were cross-sectioned through the midpoint to analyze histologic damage. Periodic acid-Schiff (PAS) staining was performed as previously described [14]. Degree of tubular damage was assessed on a scale of 1 to 4 (no histopathological findings observed, focal area of tubular necrosis involving <25% of the kidney, tubular necrosis involving 25–50% of the kidney, and tubular necrosis involving >50% of the kidney corresponding to 1–4, respectively) (N = 6) [14].

### TUNEL assay

Apoptosis was detected in paraffin sections from each group using the terminal deoxynucleotidyl transferase (TdT)-mediated uridine triphosphate (dUTP) nick end labeling (TUNEL) as previously described [15]. Sections (5 μm) were stained using *in situ* DeadEnd^TM^ Colorimetric Apoptosis Detection System (Promega, Madison, WI, USA), according to the manufacturer’s instructions. Five visual fields from each sample block were randomly selected and analyzed by a blinded observer using an Olympus microscope with 400× magnification. Apoptotic index, defined as (apoptotic cells/total cells) × 100%, was determined using 20 fields per sample (N = 6).

### BUN and creatinine analysis

BUN and serum creatinine concentrations were determined 24 h after reperfusion using picric acid and diacetyl monoxime methods [16], respectively.

### Immunoblotting

Tissue samples were lysed in RIPA buffer [10 mM Tris-HCl (pH 7.5), 1 mM EDTA, 150 mM NaCl, 1% NP-40, 2% SDS, 1% sodium deoxycholate, 50 mM NaF, 0.2 mM Na_3_VO_4_, 1 mM PMSF, and phosphatase inhibitor cocktail I and II (Sigma, St. Louis, MO, USA)]. Protein amounts were determined using the Quick Start Bradford 1× Dye reagent (Bio-Rad, USA).

Proteins were separated on sodium dodecyl sulfate-polyacrylamide gel electrophoresis (SDS-PAGE) and immunoblotted with anti-phospho-eNOS, anti-eNOS, anti-phospho-iNOS, anti-iNOS, anti-Bcl-2, anti-Bax, anti-PARP, anti-cleaved-PARP, anti-interleukin 1β, anti-phospho-AKT, anti-AKT, anti-phospho-ERK, anti-ERK, and anti-actin antibodies obtained from Cell Signaling Technology (Beverly, MA, USA), and anti-CTP1A, anti-NOX4, anti-TXNIP, anti-NLRP3, anti-ASC, anti-caspase-1, and anti-cleaved caspase-1 antibodies obtained from Abcam (Cambridge, UK) (N = 6).

### ELISA

Serum levels of IL-6 and tumor necrosis factor (TNF)-α were determined by commercial ELISA kits (R&D Systems, Minneapolis, MN, USA), according to the manufacturer’s instructions.

### Statistical analysis

Data are expressed as means ± S.D. and were analyzed using one-way analysis of variance (ANOVA), followed by Bonferroni correction or repeated measures two-way ANOVA. P-values <0.05 were considered significant.

## Results

### Hemodynamic parameters

Diabetic rats displayed increased blood glucose levels and reduced body weight. Table 1 shows hemodynamic data for all groups throughout the study period. No significant differences in MAP and HR at baseline or during ischemia were observed between groups. MAP was increased by IR; however, post-reperfusion treatment with DEX significantly attenuated this increase (p < 0.05). IR-induced HR increase was significantly attenuated in both DEX-treated groups (p < 0.05).

**Table 1:** Changes in hemodynamic parameters (means ± SD) in diabetic rats.

**MAP**	**D-Sham**	**D-IRC**	**DEX^pre^**	**DEX^post^**
**Baseline**	90.2 ± 17.7	89.4 ± 9.6	96.3 ± 12.2	90.7 ± 7.0
**During ischemia**		84.7 ± 23.1	81.1 ± 5.1	73.9 ± 11.0
**After reperfusion**		120.6 ± 7.4*	123.2 ± 8.1*	114.1 ± 4.4*^,^^‡^
**HR**	**D-Sham**	**D-IRC**	**DEX**^**pre**^	**DEX**^**post**^
**Baseline**	269.3 ± 20.8	281 ± 25.9	314.5 ± 99.7	265.4 ± 23.8
**During ischemia**		254.1 ± 71.7	230.0 ± 49.7	238.5 ± 29.8
**After reperfusion**		312.1 ± 26.8*	263.6 ± 30.5^†^	257.9 ± 27.1^†^

MAP and HR were recorded 15 min after starting intraperitoneal anesthesia (baseline), 45 min after inducing ischemia (during ischemia), and after 24-h reperfusion (after reperfusion).

MAP: mean arterial blood pressure; HR: heart rate; DEX: dexmedetomidine; D-IRC: diabetic ischemia-reperfusion control.

*p < 0.05, compared with the sham baseline

†p < 0.05, compared with D-IRC group

‡p < 0.05, compared with DEX pre-treatment group

### DEX reduced renal tubular apoptosis in diabetic rats

**Fig 1:**
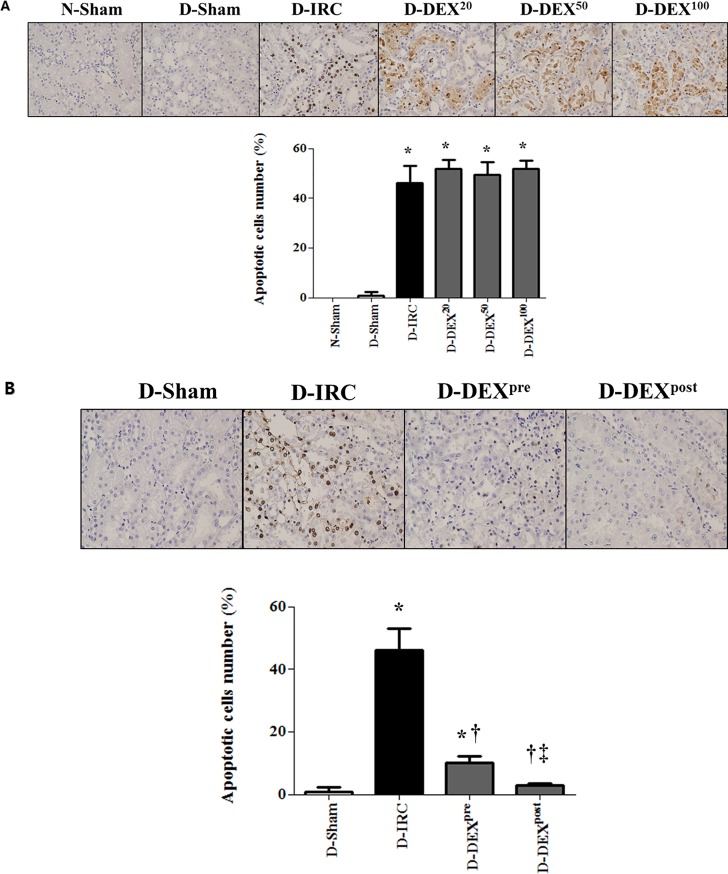
DEX reduced IR-induced renal tubular cell death in diabetic rats, TUNEL X40. High-dose DEX treatments (20, 50, and 100 μg/kg) did not reduce apoptosis (A). DEX (10 μg/kg) reduced apoptosis (B). DEX^20^, DEX^50^, and DEX^100^ correspond to DEX treatments at doses of 20, 50, and 100 μg/kg, respectively; *p < 0.05, compared with D-sham baseline; †p < 0.05, compared with D-IRC group; ‡p < 0.05, compared with DEX pre-treatment group.

No apoptotic cells were observed in the kidney tissues of the normal sham-operated group. A small number of apoptotic cells were observed in the diabetic sham-operated group; however, the difference between the sham groups was not statistically significant. IR injury significantly increased the number of apoptotic tubular cells in diabetic rats. High-dose DEX treatments (20, 50, and 100 μg/kg) did not reduce apoptosis (Fig 1A), whereas 10 μg/kg of DEX significantly ameliorated IR-induced apoptosis (p < 0.05). DEX post-reperfusion treatment group displayed significantly reduced tubular cells apoptosis, compared to DEX pre-treatment group (p < 0.05, Fig 1B).

### DEX improved renal tubular damage following IR in diabetic rats

**Fig 2:**
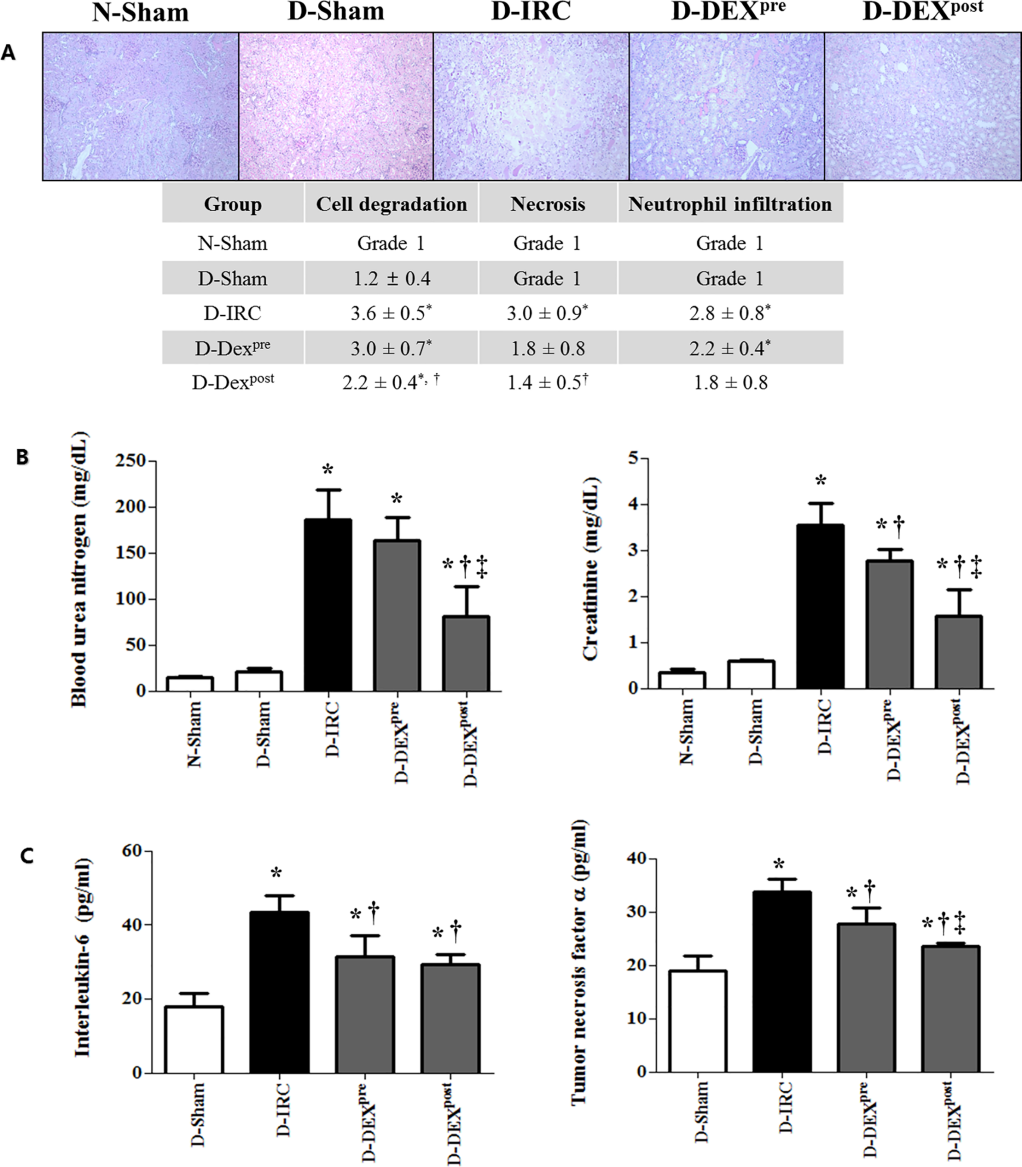
DEX improved histopathological scores and reduced markers of renal dysfunction and inflammation in renal IR injury, PAS X40. Post-reperfusion treatment with DEX improved histopathological scores of renal tubular damage in diabetic rats (A). DEX reduced BUN and creatinine (B) and IL-6 and TNF-α levels (C) in renal IR injury. *p < 0.05, compared with D-sham baseline; †p < 0.05, compared with D-IRC group; ‡p < 0.05, compared with DEX pre-treatment group.

Histopathological scores of renal tubular damage were significantly higher in the D-IRC group, than in the D-sham group (Fig 2A). Post-reperfusion DEX treatment significantly improved tubular damage compared to the D-IRC group (p < 0.05).

### Post-reperfusion treatment with DEX attenuated IR-induced renal dysfunction in diabetic rats

BUN and serum creatinine levels observed 24 h after IR were significantly higher than those measured in the D-sham group (p < 0.05). Compared to the untreated D-IRC group, post-reperfusion treatment with DEX significantly reduced BUN and creatinine levels (p < 0.05) (Fig 2B).

### DEX reduced serum levels of IL-6 and TNF-α following IR injury in diabetic rats

Following IR, serum IL-6 and TNF-α levels significantly increased in the D-IRC group compared with that in the D-sham group (p < 0.05). Pre- and post-reperfusion DEX treatments significantly decreased IR-induced elevation of IL-6 and TNF-α (p < 0.05) (Fig 2C).

### DEX did not affect expression of p-eNOS and attenuated expression of p-iNOS in diabetic rats

**Fig 3:**
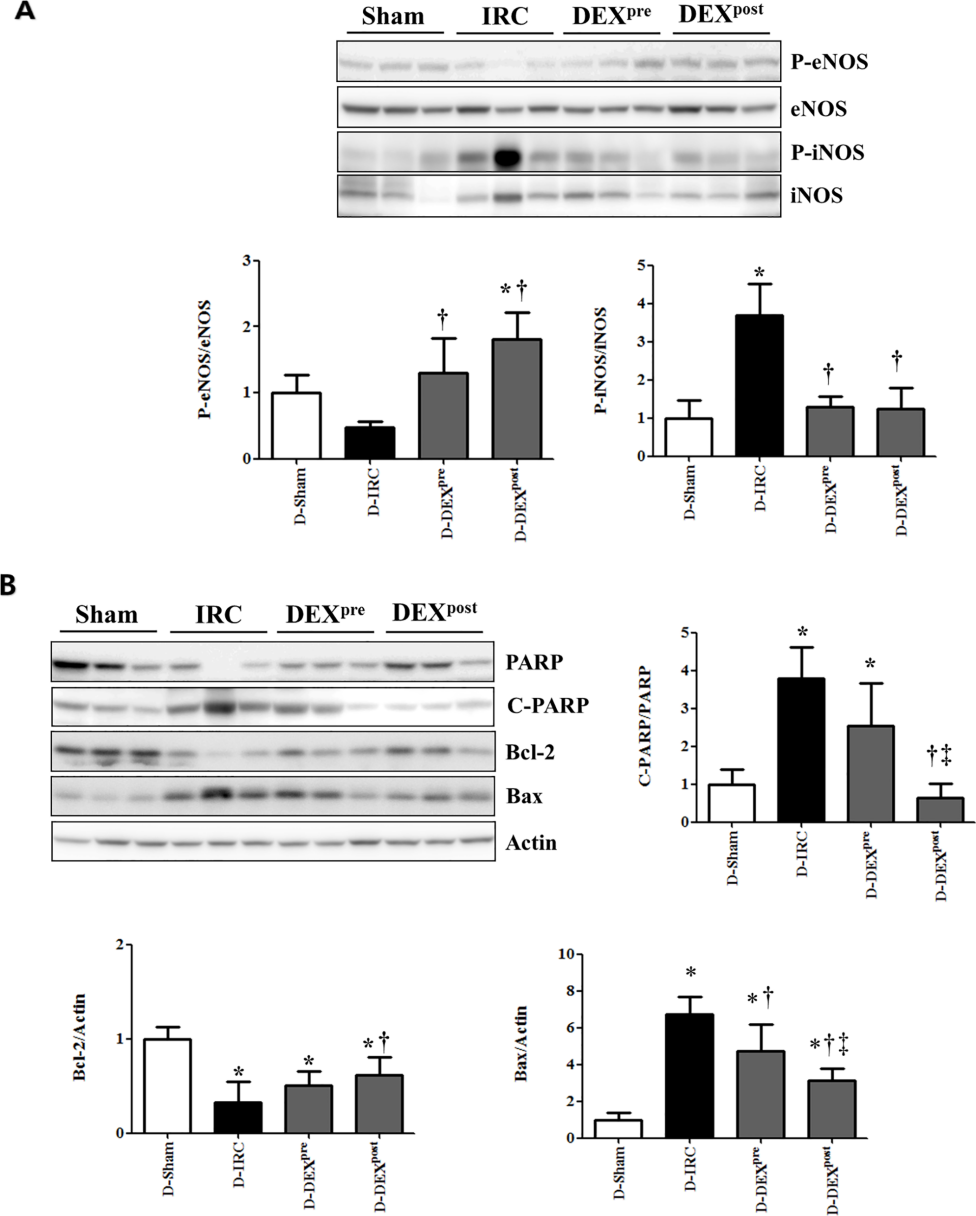
Effects of DEX on NOS, cleaved-PARP, Bcl-2, and Bax in renal IR injury. DEX did not affect the expression of p-eNOS and attenuated the expression of p-iNOS in diabetic rats (A). Post-reperfusion treatment with DEX reduced the increase in cleaved-PARP and did not affect Bcl-2 and Bax expression in renal IR injury (B). *p < 0.05, compared with D-sham baseline; †p < 0.05, compared with D-IRC group; ‡p < 0.05, compared with DEX pre-treatment group.

The levels of p-eNOS decreased in the D-IRC group compared to the levels in the D-sham group, whereas p-eNOS expression in both pre-treatment and post-reperfusion DEX treatment groups was retained. Expression of p-iNOS was barely detectable in the D-sham group and significantly upregulated in the D-IRC group (p < 0.05). Both DEX pre-treatment and post-reperfusion treatment significantly decreased p-iNOS expression compared to the expression in the D-IRC group (p < 0.05, Fig 3A).

### DEX favorably altered expression of Bcl-2, Bax, and cleaved-PARP in diabetic rats

Compared with the D-sham group, Bcl-2 expression was significantly reduced in the D-IRC group, and significantly higher in the DEX post-reperfusion treatment group than in the D-IRC group (p < 0.05). In contrast, cleaved-PARP and PARP expression significantly increased in the D-IRC group compared with that in the D-sham group, and this effect was ameliorated by DEX post-reperfusion treatment (p < 0.05) (Fig 3B). Both pre- and post-reperfusion treatment with DEX ameliorated IR-induced increase of Bax expression, compared to the D-IRC group (p < 0.05), with post-reperfusion DEX treatment proving more effective (p < 0.05).

### Post-reperfusion DEX treatment attenuated the increase in CTP1A, NOX4, and TXNIP levels following IR in diabetic rats

**Fig 4:**
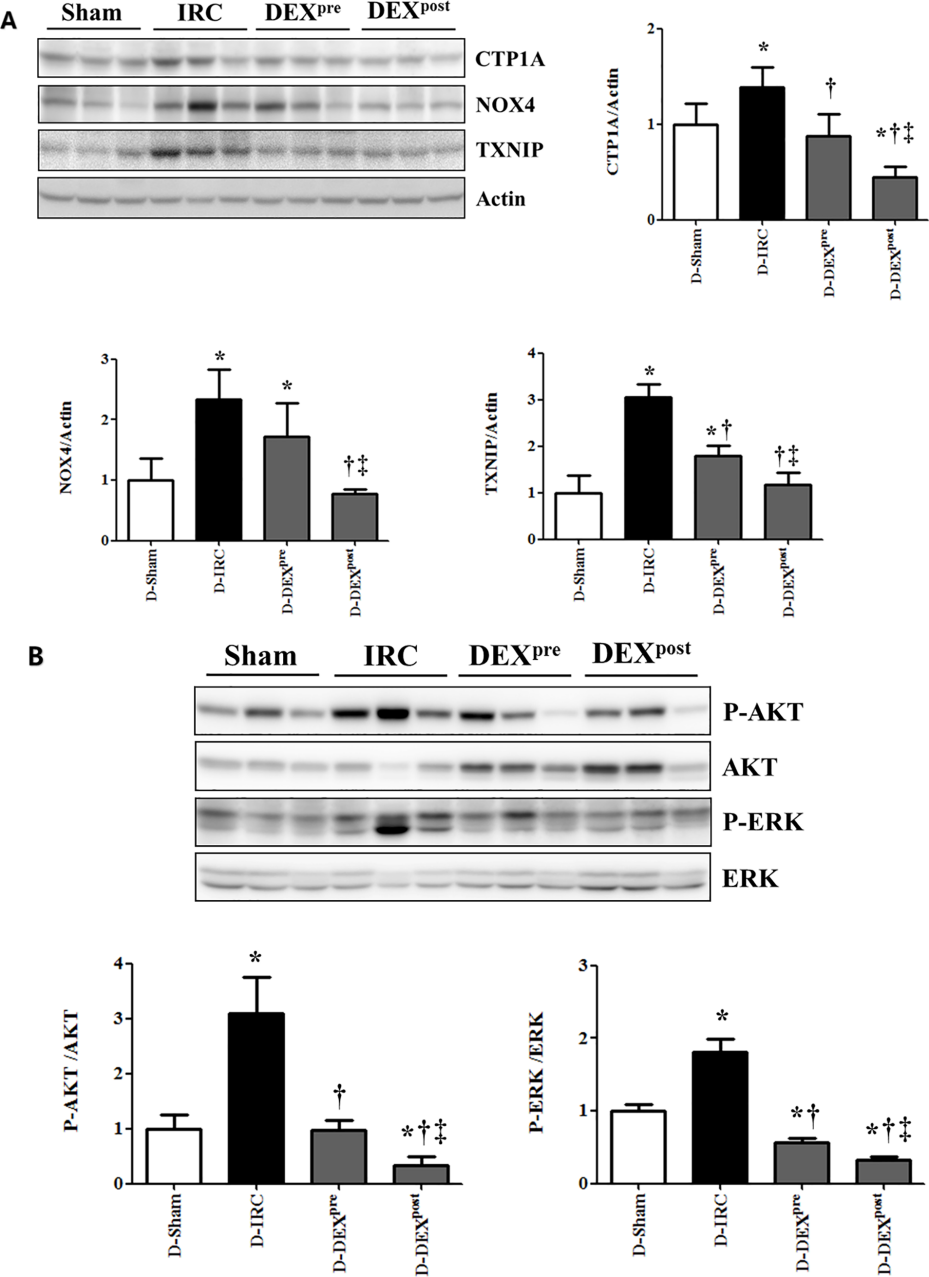
Effects of DEX on oxidative stress markers, AKT and ERK signaling in renal IR injury. Post-reperfusion DEX treatment attenuated the increase in CTP1A, NOX4, and TXNIP in renal IR injury (A) and reversed the increase in phospho-AKT and phospho-ERK levels in renal IR injury (B). *p < 0.05, compared with D-sham baseline; †p < 0.05, compared with D-IRC group; ‡p < 0.05, compared with DEX pre-treatment group.

Following IR, CTP1A, NOX4, and TXNIP levels significantly increased in the D-IRC group compared with that in the D-sham group, an effect significantly reduced in the DEX post-reperfusion treatment group (p < 0.05, Fig 4A).

### Post-reperfusion DEX treatment decreased IR-induced increase in phospho-AKT and phospho-ERK in diabetic rats

Pre- and post-reperfusion DEX treatments ameliorated IR-induced increase in phospho-AKT and phospho-ERK compared to the D-IRC group levels (p < 0.05), with post-reperfusion treatment with DEX proving more effective (p < 0.05, Fig 4B).

### Post-reperfusion DEX treatment ameliorated the increase in NLRP3, cleaved caspase-1, and IL-1β levels following IR in diabetic rats

**Fig 5:**
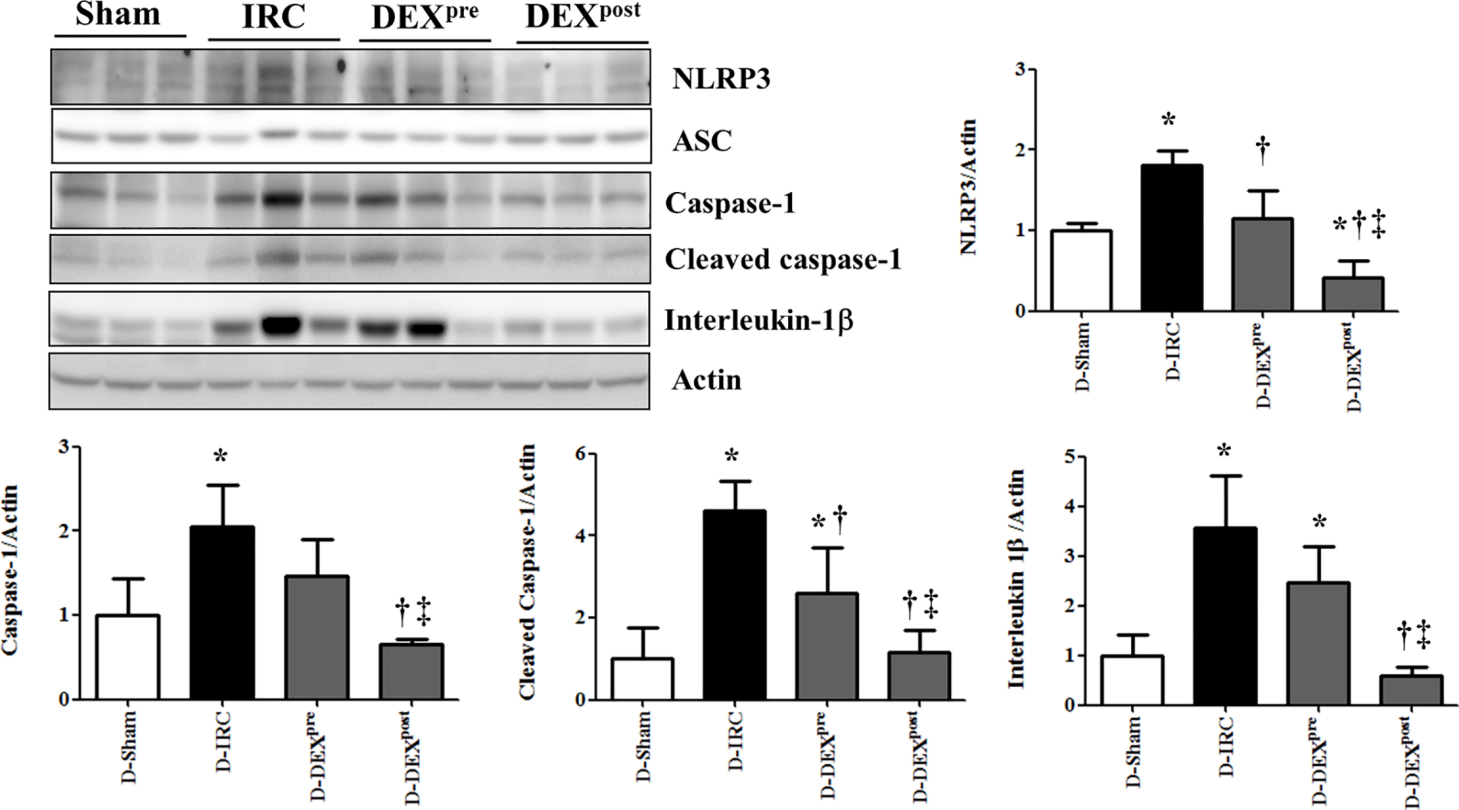
Post-reperfusion DEX treatment ameliorated the increase in NLRP3, cleaved caspase-1, and IL-1β in renal IR injury. *p < 0.05, compared with D-sham baseline; †p < 0.05, compared with D-IRC group; ‡p < 0.05, compared with DEX pre-treatment group.

IR-induced increase in the levels of NLRP3, cleaved caspase-1, and IL-1βwas significantly diminished in the DEX post-reperfusion treatment group compared with that in the D-IRC group (p < 0.05). No significant difference in ASC levels was observed between diabetic rat groups (Fig 5).

## Discussion

This is the first study to demonstrate that the renoprotective effects of DEX in renal IR injury are associated with inflammasomes in a diabetic rat model. Renal IR injury increased the levels of inflammasome components NLRP3 and caspase-1, an effect reversible by DEX. DEX also reduced increased IL-1βlevels, activated by NLRP3 inflammasome. NLRP3 inflammasome-associated protective effect of DEX was greater in the post-reperfusion treatment group than in the pre-treatment group. Post-reperfusion DEX treatment was also more effective than pre-treatment in terms of AKT and ERK signaling, oxidative stress, and renal dysfunction.

NLRP3 inflammasome contributes to the development of type 1 and 2 diabetes mellitus [17,18]. It is a complex of cytosolic proteins comprising NLRP3, apoptosis-associated speck-like protein containing a caspase recruitment domain (ASC), and caspase-1. When NLRP3 inflammasome is activated, caspase-1 and subsequently IL-1βare cleaved to their active forms, stimulating inflammatory cascades. It has been elucidated in recent studies that activation of NLRP3 inflammasome leads to the progression of various renal conditions and is recognized as a promising potential therapeutic target [3,4]. Chen et al. analyzed renal interstitial inflammation in diabetes patients and found increased levels of NLRP3, IL-1βand IL-18 in diabetic nephropathy, compared to normal controls [19]. Inhibition of NLRP3 inflammasome activation protects diabetic kidney from IR injury and diminishes sensitivity of diabetic kidney tissues to AKI [20]. DEX has shown organ protective properties against IR injury in multiple organs possibly via modulation of cell death by apoptosis, activation of cell survival kinases, and modulation of inflammatory responses and oxidative stress [8–10,21,22]. Thus, we hypothesized that inhibition of NLRP3 inflammasome might retain the renoprotective effects of DEX against renal IR injury in a STZ-induced diabetic rat model.

In this study, renal IR injury increased NLRP3, caspase-1, cleaved caspase-1, and IL-1βlevels, whereas NLRP3 inflammasome activation was suppressed by DEX treatment, indicating that protective effects of DEX are well preserved under diabetic conditions. Although DEX renoprotective effects against IR injury in diabetic rats are achieved by ameliorating lipid peroxidation and oxidative stress [8], the present study is the first to demonstrate that the protective effects of DEX against renal IR injury in diabetic rats are associated with the inhibition of NLRP3 inflammasome activation. This result is consistent with recent studies in non-diabetic animal models demonstrating that DEX reduces NLRP3 inflammasome activation [23].

Both pre-ischemia and post-reperfusion treatments of DEX ameliorated IR-induced increase in phospho-AKT, phospho-ERK, and pro-apoptotic Bax levels compared to the D-IRC group levels. DEX post-reperfusion treatment enhanced anti-apoptotic Bcl-2 activity and reduced cleaved-PARP levels compared to D-IRC group. Reperfusion injury salvage kinase pathway, which combines two parallel cascades: phosphatidylinositol-3 kinase (PI3K)-AKT and p42/p44 ERK, is an important pro-survival pathway in IR injury. Kim et al. demonstrated that activation of PI3K-AKT is accompanied by decreased anti-apoptotic Bcl-2 and increased pro-apoptotic Bax levels in *db/db* mice [24]. Increased ERK activity was observed in human and rodent diabetic adipose tissue, and inhibition of the ERK pathway is a potential therapeutic strategy to combat insulin resistance [25]. Reactive oxygen species (ROS) produced during IR injury trigger over-activation of the nuclear enzyme poly (ADP-ribose) polymerase (PARP), which induces translocation of apoptosis-inducing factors, causing cell death [26]. DEX attenuates apoptosis by inhibiting the activation of intrinsic apoptotic cascades [27].

In diabetes, hyperglycemia can activate ROS and NLRP3 inflammasome. During IR, ROS promote tissue inflammation and activate immune responses through different pathways, including NLRP3 inflammasome [28]. Previously, TXNIP-mediated NLRP3 activation via oxidative stress was identified as a key signaling mechanism in susceptibility to AKI [20,29]. DEX administered to diabetic rats ameliorates lipid peroxidation, oxidative stress, and IR-related renal injury partly by inhibiting P38-MAPK (mitogen-activated protein kinases) activation and expression of TXNIP in diabetic kidney [8,30]. In this study, DEX treatment, especially post-treatment, attenuated increase in oxidative stress markers (CTP1A, NOX4, and TXNIP) following IR. Furthermore, increased inflammatory markers such as IL-6 and TNF-α in IR injury, as expected, were diminished by DEX. DEX was reported to decrease the production of cytokines, including IL-1βand TNF-α, in renal IR injury model [31].

Nitric oxide synthases (NOS), a family of enzymes catalyzing the production of NO, contribute to cellular damage during IR injury. Different types of NOS differently affect cell viability. NO produced by endothelial NOS (eNOS) has antioxidant properties as it reduces superoxide anion formation, whereas up-regulation of inducible NOS (iNOS) is associated with oxidative stress cytotoxicity. Increased iNOS activity is dependent on the activation of ERK-mediated phosphorylation [32]. In this study, pre-ischemia and post-reperfusion DEX treatments retained phospho-eNOS and decreased phospho-iNOS expression compared to the D-IRC group.

In this study, DEX treatment after IR injury improved renal protection compared to preemptive administration. Therefore, this study provides evidence on retained renoprotective efficacy of DEX under diabetic conditions against IR injury and demonstrates a large therapeutic window of these effects relative to the onset of ischemic insult. However, the optimal timing of DEX administration remains controversial [6,33]. Gonullu et al. showed that DEX administered before ischemia and after reperfusion histomorphologically reduced renal IR injury, with administration of DEX during reperfusion considered more effective [6]. ROS generation is markedly increased immediately after reperfusion, reaches its peak after 4 to 7 minutes, and is maintained thereafter, resulting in oxidative stress. In human volunteers, intravenous administration of clinical dose of DEX exhibits a rapid distribution phase with a distribution half-life of approximately 6 minutes and an elimination half-life of 2.1–3.1 h. In rats with single infusion of 30 μg/kg for 10 minutes, pharmacokinetic characterization showed a distribution half-life of approximately 2 minutes and a terminal half-life of 57 minutes [34]. The superiority of post-reperfusion DEX treatment over pre-ischemia DEX treatment in this study might be related to the pharmacokinetics of DEX and pathophysiology of renal IR injury in diabetic conditions. Further research is needed to investigate the optimal timing of DEX administration in aspects of relationship between DEX plasma concentrations and renal effects.

In previous studies of rodent models, a wide range of DEX intraperitoneal doses (10–100 μg /kg) showed renoprotective effects against renal IR injury [5–7,31]. However, no clear recommendations regarding the effective DEX dose ranges in renal IR injury have been established in diabetic model. In this study, we determined the appropriate dose of DEX for renal protection on the basis of preliminary data testing various DEX doses (10, 20, 50, and 100 μg/kg).

## Conclusions

This study showed that the protective effects of DEX in renal IR injury are preserved under diabetic conditions and are related to inflammasomes. Furthermore, post-reperfusion DEX treatment was more effective than pre-ischemia treatment. These findings potentially provide a basis for using DEX in treating renal IR injury.

## Supporting information

S1 Table Original data. This file contains the original data in the figures.
(XLSX)
